# The role of gonadal hormones in regulating opioid antinociception

**DOI:** 10.1080/07853890.2024.2329259

**Published:** 2024-05-13

**Authors:** Qi Xu, Lin Jin, LuYang Wang, YingYing Tang, Hui Wu, Qing Chen, LiHong Sun

**Affiliations:** Department of Anesthesiology, Women’s Hospital, School of Medicine, Zhejiang University, Hangzhou, Zhejiang Province, China

**Keywords:** Gonadal hormones, pain, opioid, oestrogen, testosterone, progesterone

## Abstract

Opioids are the most prescribed drugs for the alleviation of pain. Both clinical and preclinical studies have reported strong evidence for sex-related divergence regarding opioid analgesia. There is an increasing amount of evidence indicating that gonadal hormones regulate the analgesic efficacy of opioids. This review presents an overview of the importance of gonadal steroids in modulating opioid analgesic responsiveness and focuses on elaborating what is currently known regarding the underlyingmechanism. We sought to identify the link between gonadal hormones and the effect of oipiod antinociception.

## KEY MESSAGES

1. Gonadal hormones contribute to the sexual dimorphism of opioid antinociception.
2. Generally, oestradiol is a negative modulator of opioid analgesia via both non-genomic and genomic effects.
3. Testosterone facilitates opioid analgesia mainly through the transcriptional activities of androgen receptors.
4. Under normal physiological conditions, progestin and oestrogen exist in parallel and have a combined effect. However, progestin alone could promote opioid analgesia by increasing the expression of opioid receptors.

## Introduction

Sex differences in pain involve many aspects, including the types and frequency of pain syndromes, the prevalence and severity of pain, pain control and responsiveness to analgesics [1,2]. Women are at substantially higher risk than men of several clinical pain conditions, such as migraine, irritable bowel syndrome, interstitial cystitis and chronic pelvic pain [3,4]. Apart from epidemiological differences, numerous studies have reported distinct sex-related divergence in the efficacy of opioid agonists. Among these studies, the findings of the majority suggested that lower pain thresholds and decreased antinociceptive effects of opioid agonists were observed among females in comparison to males [5]. In a clinical study, it was reported that women required a 30% higher dose of morphine to achieve a homologous degree of analgesia than men [6]. Most animal studies have confirmed that opioids produce a greater degree of analgesia in male rodents than in female rodents [7,8]. The sex-biased response to opioid analgesia may be attributed to many factors. For instance, discrepant psychological mechanisms are thought to play a fundamental role, with men and women tending to use different coping methods to manage pain [9]. In addition, there are differences in the endogenous opioid functioning system between men and women in the activation of μ-opioid receptors (MOR) in the central nervous system [10]. Furthermore, different circulating levels of gonadal hormones were also reported to play major roles in mediating the sexual dimorphism of opioid antinociception. The current review focuses on elaborating the regulatory role of gonadal steroids in the sex-biased response to opioids.

## The effect of gonadal steroids on opioid-mediated analgesia

Gonadal steroids are hormones produced by the gonads (ovaries and testes). The ovaries primarily produce oestrogens and progestins, and the principal products of testes are androgens. Gonadal hormones have multivariate impacts on reproduction, endocrine function, metabolism, immunity and the function of the central nervous system [11,12]. Cyclical changes in the levels of endogenous sex hormones in women create the follicular (pre-ovulation) and luteal (post-ovulation) phases of the menstrual cycle. Under the influence of follicle-stimulating hormone (FSH), the secretion of oestrogen increases rapidly, with the first peak of oestrogen appearing in the early stage of ovulation. The secretion of oestrogen also promotes the secretion of FSH and luteinizing hormone (LH) through a positive feedback effect on the hypothalamus and pituitary gland, forming the LH peak and promoting the ovulation of mature follicles. After ovulation, the body enters the luteal phase, during which the luteal body promotes the secretion of oestrogen, leading to the second peak of oestrogen after ovulation (this peak is lower than the first peak). The peak of oestrogen promotes the luteal body to secrete progestin, which peaks 5 to 10 days after ovulation [13,14]. The influence of sex hormones represents a significant source of variability in response to opioids [15,16]. The effect of opioid analgesia was significantly greater in the follicular phase than in the luteal phase [17,18]. However, the results are not always consistent. Ahmed et al. found no significant difference in morphine needs between the luteal phase and the follicular phase [19]. For males, it is commonly believed that androgen facilitates morphine analgesia. Many studies have confirmed that hypogonadism usually increases the dose and frequency of opioid use, which can typically be reversed by testosterone treatment [20,21]. There are also several exceptions to these findings. For example, Huang et al. reported that testosterone replacement had no effect on the requirement of ­opioid intake [22,23].

Overall, in the preclinical literature, different approaches have been adopted to examine the modulation of opioid analgesia by gonadal steroids. Some of the studies compare the efficacy of opioids between male and female animals: An abundance of data suggests that male rodents are usually more sensitive than female rodents to the antinociceptive actions of both MOR and κ-opioid receptor (KOR) agonists, which were administered both systemically and centrally [24,25]. Some studies have compared opioid analgesia effects at different phases of the oestrous cycle in female animals. The oestrous cycle of normal female animals is divided into proestrus, oestrous, metestrus and diestrus. Serum oestrogen levels are highest in proestrus and lowest at the beginning of diestrus. The luteal body secretes progestins during the metestrus period, causing a higher progesterone level in metestrus than in other phases [26]. The extent of morphine analgesia was greatly influenced by changes in hormone levels at different time points of the oestrous cycle (Table 1). It was reported by Stoffel et al. that in female rats, the analgesic effect of morphine administered by subcutaneous injection during the oestrous phase was lower than that observed during diestrus or proestrus [27]. Morphine administered intraperitoneally was more potent in metestrus and proestrus than in oestrous [28]. It was recently demonstrated that female rats in diestrus had a higher analgesic effect of oral administration of oxycodone, an agonist of both κ and δ opioid receptors, than those in oestrous [29]. Kosiorek et al. reported that the sensitivity of female rats to subcutaneous administration of morphine in the diestrus and oestrous phases was higher than that in the metestrus phase [30]. The antinociceptive efficacy of morphine microinjected into the ventrolateral periaqueductal grey (PAG) was found to be highest in female diestrus phase of rats [31]. In general, most literature concluded that the antinociceptive effect of opioid was higher during diestrus, when the level of circulating oestrogens and progestins is the lowest, than other stages. However, there are some contradictory findings. For example, Liu et al. found that during diestrus of rats, the antinociceptive responsiveness of intrathecal application of endomorphin-2 (EM2), was lower than that during proestrus stage [32]. Furthermore, Escudero et al. reported that the analgesic effect of subcutaneous injection of U50,488H, a κ opioid receptor agonist, was comparable in all cycle phases of mice [33]. The discrepancy in these results may be explained by the fact that the physiological activity of hormones in different phases of the oestrous cycle is not simply determined by hormone levels at that time of the cycle. For example, in early oestorus, although the oestrogen level is lower than in proestrus, the prolonged effect of the high oestrogen level from the previous stage on opioid analgesia might still persist. Therefore, in addition to hormone levels, changes in opioid analgesic effects are more related to the paradigm of hormone fluctuations in the body [34]. Some studies in the literature have compared opioid analgesia between gonadally intact and gonadectomized animals. Most studies concluded that gonadectomy (GDX) decreased opioid antinociception in male animals and increased opioid effects in female animals [35,36]. There were some contradictory studies reporting that opioid analgesia was not altered by gonadectomy [23,37]. Some laboratories have analysed the analgesic effect in GDX animals with or without hormone replacement. The efficacy of morphine has been reported to be decreased [38] or unchanged [39] after exposure to oestradiol or progesterone replacement therapy in ovariectomized (OVX) female rats. Many studies reported that the antinociceptive effect of opioids in adult male rats after castration was increased by testosterone administration [35,40]. However, Peckham et al. reported no effect of testosterone replacement on opioid antinociception [23].

**Table 1.** Comparison of opioid analgesia in different stage of oestrous cycle.

Species	opioid and route	Reported findings	Reference
SD rats	s.c. morphine	D,P > E	[27]
F344, Lewis, Long Evans, and Wistar rats	i.p. morphine	M, P > E	[28]
Wistar rats	oral oxycodone	D > E	[29]
SHR rats	s.c. morphine	D,E > M	[30]
SD rats	microinjection of morphine into PAG	D > P, E	[31]
SD rats	i.t. EM2	D < P	[32]
C57BL/6J mice	s.c. U50,488H	comparable in all cycle phases	[33]

SD: Sprague Dawley; s.c.: subcutaneous; D: diestrus; P: proestrus; E: oestrous; M: metestrus; i.p.: intraperitoneal; SHR: spontaneously hypertensive rat; PAG: ventrolateral periaqueductal grey; EM2: endomorphin-2.

The discrepancy in the effects that ovarian steroids exert on opioid systems may be attributed to discrepancies in the genotype of animal species, the receptor type of the opioid agonist used, the dose and efficacy of opioid agonists, the route and time of opioid administration, and the modality and intensity of nociceptive stimuli [5]. For example, a study found that the efficacy of opioids with diverse receptor selectivity was differentially modulated by gonadal hormones: GDX decreased morphine (MOR agonist) analgesia but had no effect on U50488 (KOR agonist) antinociception in male rats. OVX enhanced morphine analgesia but did not alter the efficacy of U50488 in female rats [41]. This indicates that the effects of gonadal steroids on opioid analgesia are related to the receptor type. How do gonadal steroids regulate opioid antinociception? The signalling crosstalk between different gonadal hormones and the opioid system is intricate. Hereinafter, we focus on illuminating the cellular and molecular mechanisms of the sex-based differences in opioid analgesia mediated by different steroid hormones, including oestrogen, androgen and progestin.

## The role of oestrogen in the sexual dimorphism of opioid antinociception

Oestrogens mainly comprise oestradiol (E2), oestrone (E1) and oestriol (E3). Under the action of FSH and LH, the ovaries synthesize oestrogen from low-density lipoprotein cholesterol [42]. E2 is the predominant natural oestrogen in females. It comes mainly from the ovaries but can also be produced locally in the brain by aromatase catalysing cholesterol or androgens. The biological function of E2 is embodied as a key regulatory factor in the body’s reproduction, differentiation, cell proliferation, inflammation, and metabolism [43]. In addition to its well-established effects on female reproductive functions, E2 exerts various actions on the nervous system, influencing pain sensation, mood, and cognitive function [11].

E2 alters opioid analgesic effects by regulating the function of cytochrome P450 2D (CYP2D), which is critical in the metabolism of opioid agonists. E2 increases CYP2D to cause a lower oxycodone concentration in the brain, resulting in lower analgesia effects of orally administered oxycodone [29]. In addition to regulating opioid metabolism, E2 is also known to change endogenous opioid neurotransmission and acts as a major determinant of opioid receptor functionality [44]. There exists a sex-related differential component of opioid receptors that were activated after spinal application of morphine. In both sexes, MOR is predominant in inhibiting pain. In spinal morphine analgesia, the involvement of theKOR is female-specific [45,46]. In the spinal cord of rats, the heterodimerization of MOR and KOR, which has been thought to adjust the antinociceptive function of spinal KOR activation, is more prevalent in proestrus (when circulating oestrogen level is high) than in diestrus or in males [47]. This female-specific and high oestrogen-level stage-dependent formation of MOR/KOR heterodimers strongly suggests that oestrogen is the internal driving force of MOR/KOR heterodimerization [48]. OVX could facilitate KOR-mediated analgesia in female mice, which could be reversed by intraperitoneal administration of 17β-oestradiol. E2 stimulates the phosphorylation of G-protein coupled receptor kinase 2 (GRK2) at the S670 site to increase Gβγ sequestration, leading to the inhibition of G protein-coupled receptor (GPCR) signalling both in the ventral striatum and in the spinal cord of mice. In this way, E2 blunts the analgesic effect of intraperitoneal application of U50488, a selective KOR agonist [36]. In contrast, Lawson et al. found that the antinociception and anti-hyperalgesia produced by intrathecal administration of U50488H in rats were enhanced by subcutaneous oestradiol administration by upregulating the expression of the KOR gene [49]. This inconsistency in KOR agonist-mediated analgesia may be due to discrepancies in the mode and dose of drug administration, species differences, or differences in oestrogen levels between study subjects. Oestrogen negatively modulates the analgesic responsiveness of spinal EM2, the predominant form of endogenous μ opioid ligand, in female rats [50]. Glutamate-activated metabotropic glutamate receptor 1 (mGluR1) and concomitant mGluR2/3 activation are critical to form a responsive state of EM2 antinociception in rats. Oestrogen shifts the activator of mGluR1 from glutamate to oestrogen receptor α (ERα), resulting in the suppression of EM2 analgesic responsiveness. ERα/mGluR1 signalling, activated by oestrogen, impairs the EM2 analgesic effect by inhibiting dynorphin release, which is a prerequisite for EM2 antinociception [51].

The linkage of G-protein-coupled receptors to the effector system can be mediated by E2. In intact female rats, intrathecal administration of orphanin FQ, the activator of opioid receptor-like 1 receptor (ORL1R), failed to produce antinociception during the phase of proestrus. E2 diminishes orphanin FQ-induced antinociception in response to changes in endogenous oestrogen levels during different estrous cycles [52]. This oestrogen-related reduction in ORL1R-mediated analgesia was mediated by the activation of membrane oestrogen receptors (GPR30, G protein-coupled receptor 30; ERα, oestrogen receptor α; Gq-mER, G protein-coupled receptor membrane oestrogen receptor) and downstream changes in mitogen-activated protein kinase (MEK)/activated extracellular signal-regulated kinase 2 (ERK2). The coupled activation of MOR and the G protein-regulated inward rectifying potassium channel (GIRK) is required for MOR-mediated hyperpolarization of the membrane [53]. ERK2 phosphorylates ORL1R to both decrease channel activity and decrease its coupling to the GIRK channel, resulting in the inhibition of the hyperpolarization of dorsal horn neurons [54]. A study conducted on hypothalamic arcuate nucleus neurons dissected from OVX female Topeka guinea pigs showed that acute E2 treatment could reduce the coupling of MOR to its respective effector, leading to the inhibition of the response of (D-Ala^2^-N-Me-Phe^4^-Gly^5^-ol)-enkephalin (DAMGO), a selective MOR agonist. The E2-mediated reduction in MOR/effector coupling occurred *via* the activation of the cyclic adenosine monophosphate (cAMP)-dependent protein kinase A (PKA) pathway [55]. In addition, E2 can activate both PKA and protein kinase C (PKC) to induce heterologous desensitization of MOR in hypothalamic neurons, causing an attenuation of DAMGO efficacy [56,57]. Acute E2 exposure decreases the coupling of MORs to GIRK channels in hypothalamic proopiomelanocortin and dopamine neurons, causing a reduction in the efficacy of MOR agonists that activate these channels. This action of E2 can be blocked by inhibitors of phospholipase C, PKA, and PKC [58]. E2-induced desensitization of MOR is mediated by the activation of the G protein-coupled oestrogen receptor (GPER), which triggers rapid calcium release and in turn stimulates the translocation of PKC isoforms (α and ε) to the plasma membrane, leading to MOR phosphorylation [59].

The analgesic effect of opioids can be mediated by the endogenous descending pain modulatory circuit, which consists of the midbrain PAG-rostral ventromedial medulla (RVM)-spinal dorsal horn (SDH) [60]. The PAG projects to the RVM and in turn projects to the SDH neuron, comprising a neuronal pathway. The PAG and PAG-RVM output neurons contain a high density of MORs [61]. The disinhibition theory supports that the activation of MOR *via* opioid administration disinhibits the inhibitory effect of MOR^+^ neurons on PAG-RVM glutamatergic neurons, resulting in pain alleviation [62]. It is noteworthy that there is a physiological/anatomical sex difference regarding the quantitative expression of MORs in the PAG-RVM circuit. The higher density of MORs in the PAG-RVM pathway in males than in females contributes to the behaviour result that males have a greater antinociceptive effect of morphine administered by microinjection into the RVM than females [31]. Apart from the inherent anatomical difference, the dimorphic response to morphine can also be regulated by different fluctuating oestrogen levels. Given that MOR and ERα are colocalized in opioid-sensitive PAG–RVM output neurons, oestrogens may differentially mediate opioid efficacy in males and females by regulating the functionality of opioid receptors in the PAG-RVM ­circuit [63]. It was recently confirmed by Jiao et al. that GPER and MOR were coexpressed in the ON cells of the RVM. E2 applied intracisternally or directly into the RVM attenuated morphine analgesia *via* the ­activation of GPER, which depolarized ON cells by promoting calcium/PKC-mediated MOR phosphorylation [64].

E2 is widely known to activate its nuclear receptors ERα and ERβ to exert extensive influences on gene transcription. In the spinal trigeminal nucleus caudalis of rats, the majority of ERα- and ERβ-containing neurons express ORL1R. Oestrogen impairs ORL1R-related antinociception by inhibiting the gene expression of ORL1R in the neurons of the spinal trigeminal nucleus caudalis [65,66]. In addition, our recent research has found that in a capsaicin-induced uterine cervix pain model of OVX female rats, chronic E2 replacement impairs morphine analgesia and increases the mRNA expression of the L-type voltage-gated calcium channel Cav1.2 (α_1_C) in the spinal cord. Spinal knockdown of α_1_C could significantly reverse the oestradiol-mediated suppression of morphine antinociception [67].

**Figure 1.**
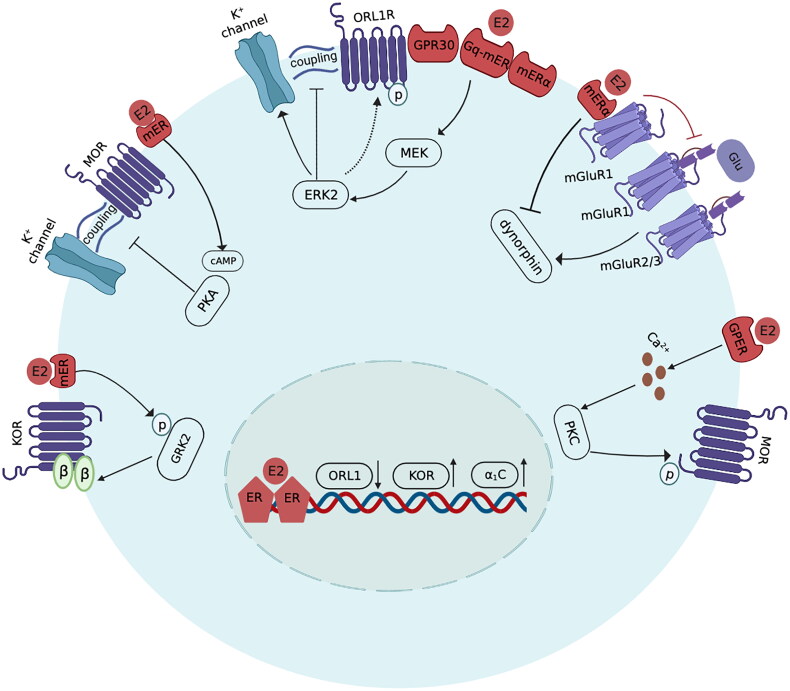
Schematic mechanisms of the intracellular signalling pathway that mediates the E2-induced attenuation of opioid analgesia. (1) E2 stimulates the phosphorylation of G-protein-coupled receptor kinase 2 (GRK2) to increase Gβγ sequestration, leading to the inhibition of GPCR signalling. (2) E2 activates cAMP-dependent PKA to reduce the coupling of MOR to its effector K^+^ channel. (3) E2 activates membrane oestrogen receptors, including Gq-mER, mERα and GPR30, to activate MEK/ERK2. ERK2 phosphorylates ORL1R to decrease both channel activity and its coupling to the GIRK channel. (4) E2 shifts the activator of mGluR1 from glutamate to ERα and reduces the binding of glutamate to mGluR1 and mGluR2/3, thereby inhibiting dynorphin release. (5) E2 activates GPER to trigger the calcium/PKC pathway, leading to MOR phosphorylation. (6) The genomic mechanisms of E2 in opioid analgesia. E2 downregulates ORL1R gene expression and upregulates Cav1.2 (α_1_C) gene expression. (E2: oestradiol; GRK2: G-protein coupled receptor kinase 2; GPCR: G protein coupled receptor; KOR: κ opioid receptor; cAMP: cyclic adenosine monophosphate; PKA: protein kinase A; MOR: μ opioid receptor; ORL1R: opioid receptor-like 1 receptor; GPR30: G protein-coupled receptor 30; mERα: membrane oestrogen receptor α; Gq-mER: G protein-coupled receptor membrane oestrogen receptor; MEK: mitogen-activated protein kinase; ERK2: activated extracellular signal regulated kinase 2; GIRK: G-protein coupled inwardly rectifying potassium; Glu: glutamate; mGluR, metabotropic glutamate receptor; GPER: G protein-coupled oestrogen receptor; PKC: protein kinase C.)

In conclusion, oestrogen could negatively regulate the efficacy of opioid analgesia by promoting the metabolism of opioids, inhibiting the functionality of opioid receptors, reducing the coupling of G-protein coupled receptors to the effector system, and ­modulating the gene expression of particular molecules closely involved in opioid signal transduction (Figure 1) (Table 2).

**Table 2.** Gonadal hormone modulation of opioid analgesia.

Species	opioid route	hormone	Analgesia test	Reported findings	Procedure notes	Citation
Wistar rats	oral oxycodone	E	tail-flick latency	Oestradiol increases cytochrome P450 2D (CYP2D) in the brain, resulting in a lower concentration of brain oxycodone and lower analgesia.	male versus female in estrus and diestrus stage, OVX female versus OVX + E female (estrogen replacement on OVX female animals)	[29]
C57BL/6N mice	i.p. U50488	E	tail withdrawal test, hot plate test	Oestradiol blunts G protein-mediated signal through increasing the phosphorylation of GRK2 and possible sequestration of Gβγ by GRK2.	male versus female, OVX female versus OVX + E female, female estrus versus non-estrus stage	[36]
SD rats	i.t. U50,488H	E	tail flick assay	Oestrogen enhances KOR analgesia *via* upregulating KOR gene expression in the spinal cord.	OVX female versus OVX + E, GDX male versus GDX + E male, female diestrus versus proestrus stage	[49]
SD rats	i.t. EM2	E	tail flick latency	Spinal mERα-mGluR1 signalling, activated by spinally synthesized oestrogen, suppresses EM2 analgesia during diestrus stage.	female diestrus versus proestrus stage	[50]
SD rats	i.t. EM2	E	tail flick latency	The association of mGluR1 with mGluR2/3 is reduced in female diestrus stage. ERα/mGluR1 signalling inhibits dynorphin release.	female diestrus versus proestrus stage	[51]
SD rats	i.t. OFQ	E	tail flick test	Oestradiol-mediated activation of mERs (GPR30, Gq-mER, ERα) could rapidly attenuate ORL1R-induced spinal antinociception *via* an ERK-dependent, non-genomic mechanism.	male versus OVX female, intact male versus intact + E male (Estrogen replacement on intact male animals), OVX female versus OVX + E female	[54]
guinea pigs	DAMGO perfusion	E	electrophysiology	Oestradiol reduces MOR analgesia *via* decreasing the coupling of MOR to its effector through cAMP/PKA pathway.	hypothalamic arcuate nucleus from OVX female versus OVX + E female	[55]
guinea pigs	DAMGO perfusion	E	electrophysiology	Oestradiol activates both PKA and PKC to cause a heterologous desensitization of MOR.	hypothalamic neurons from OVX female versus OVX + E female	[56]
human neuroblastoma SH-SY5Y cells	none	E	calcium imaging	E2/GPER triggers rapid calcium release and in turn stimulates translocation of PKC isoforms (α and ε) to the plasma membrane, leading to MOR phosphorylation	activation of GPER by E2 and G-1 (GPER selective agonist)	[59]
SD rats	microinjection of OFQ into the medullary region	E	nociceptive scratching behavior	Oestrogen impairs ORL1R-related antinociception *via* inhibiting the gene expression of the ORL1R in the neurons of spinal trigeminal nucleus caudalis.	male versus female proestrus stage, OVX female versus OVX + E female	[65,66]
SD rats	i.p. morphine	E	visceral pain score	Oestradiol impairs morphine analgesia *via* upregulating the expression of Cav1.2 in the spinal cord.	OVX female versus OVX + E female	[67]
SD rats	i.m. DAMGO	T	Von Frey filament	Testosterone reversed the GDX-induced inhibition of MOR mRNA expression in the trigeminal ganglia.	GDX male vs GDX + T male (testosterone replacement on GDX male animals)	[72]
SD rats	i.m. DAMGO	T	Von Frey filament	Testosterone and androgen receptor complex enhance the transcription of MOR gene in the trigeminal ganglia.	male vs androgen receptor antagonist-treated	[73]
Wistar rats	none	P	none	Progesterone causes an increase of DOR expression in the spinal cord.	intact male versus intact + P male (Progesterone replacement on intact male animals)	[84]
SD rats	none	P	Von Frey and Choi test	Progesterone increased KOR expression in the dorsal spinal cord.	intact male versus intact + P male	[85]

E: oestrogen; OVX: ovariectomy; i.p.: intraperitoneal; GRK2: G-protein-coupled receptor kinase 2; SD: Sprague Dawley; i.t.: intrathecal; KOR: κ opioid receptor; GDX: gonadectomy; mERα: membrane oestrogen receptor α; mGluR1: metabotropic glutamate receptor 1; EM2: endomorphin-2; OFQ: orphanin FQ; mERs: membrane oestrogen receptors; GPR30: G protein-coupled receptor 30; Gq-mER: G protein-coupled receptor membrane oestrogen receptor; ERK: extracellular signal regulated kinase; DAMGO: (D-Ala^2^-N-Me-Phe^4^-Gly^5^-ol)-enkephalin; MOR: μ opioid receptor; cAMP: cyclic adenosine monophosphate; PKA: cAMP-dependent protein kinase A; PKC: protein kinase C; GPER: G protein-coupled oestrogen receptor; ORL1R, opioid receptor-like 1 receptor; Cav1.2, voltage-gated Ca^2+^ channel 1.2; i.m.: intramuscular; T: testosterone; P: progesterone; DOR: δ opioid receptor.

## The influence of androgen on opioid-induced analgesia

Androgens, mainly testosterone (TT) and dihydrotestosterone (DHT), are responsible for the development of normal male sexual characteristics. Synthesized by testicular stromal cells and the adrenal cortex, TT is the dominant form of androgen. The production of TT is regulated by the hypothalamic-pituitary axis under the direction of gonadotropin-releasing hormone and LH. DHT is converted by TT under the catalysis of 5-a reductase [68]. Apart from maintaining normal male sexual characteristics, androgens exert biological functions, including regulating cell metabolism, neuronal proliferation, and neuronal excitability [69].

TT acts as a key regulator of the sexual dimorphism of pain perception and opioid analgesia. A high-level state of TT for males could facilitate opioid analgesia, allowing a reduction in the dose and frequency of opioid use [35]. In a persistent myogenic orofacial pain model, there was a sex difference in the efficacy of peripheral administration of the MOR agonist DAMGO between male and female rats. Compared with intact male rats, a 10-fold higher dose of DAMGO was needed to produce the same anti-allodynia level in female rats and GDX male rats due to their low levels of TT [35]. The ventrolateral periaqueductal gray (vlPAG) may be a site at which TT interacts with opioids to regulate pain inhibition. The analgesic effect of morphine *via* microinjection into the vlPAG was reduced among castrated male rats, while in androgenized female rats, the analgesic efficacy was enhanced [70].

Opioid analgesia in males mainly depends on the presence of TT during the organizational period of development, which is also known as the organizational effect of testosterone. Cicero et al. found that in female rats, large replacement doses of TT during the neonatal stage increase the antinociceptive effects of morphine in adulthood. Compared with intact male rats, GDX early in prenatal life reduced the analgesic effect of morphine administered subcutaneously. However, morphine antinociception did not change if GDX was performed in adulthood in male rats [37]. Moreover, in a carrageenan-induced persistent inflammatory pain model, subcutaneous morphine was less effective than vehicle treatment in male neonatal GDX rats. However, morphine analgesia was comparable among groups of rats gonadectomized in adulthood [71]. These findings indicate that the interaction between TT and opioid signalling is likely established during the period of sexual differentiation.

In complete Freund’s adjuvant (CFA)-induced muscle inflammation, TT replacement reversed the GDX-induced inhibition of MOR mRNA expression in the trigeminal ganglia of male rats, showing that TT plays an important role in maintaining the peripheral MOR system [72]. TT upregulates the expression of MOR in trigeminal sensory neurons *via* the transcriptional activities of androgen receptors (ARs). ARs are widely expressed in MOR-positive neurons in rat trigeminal ganglia and function as transcriptional regulators of MOR gene activities. AR activity blockade inhibits MOR expression to inhibit MOR analgesia [73]. In GDX male rats, an injection of formalin into the temporomandibular joint induces nociceptive behaviours, which can be reversed by TT replacement. This protective effect of TT on the development of nociception was mediated *via* the coactivation of central MOR and KOR [74]. A study conducted by Claiborne et al. demonstrated that intrathecal microinjection of orphanin FQ failed to produce analgesia in GDX male rats, whereas TT replacement restored the antinociceptive effect of orphanin FQ. TT might enhance ORL1R-mediated antinociception in males by upregulating ORL1R gene expression or enhancing the coupling of ORL1R to G proteins in the spinal cord [52] (Table 2).

Morphine tolerance, which is defined as a weakened response to chronic morphine exposure, can be regulated by TT. In a rat model of morphine tolerance, the serum TT level was decreased, leading to the downregulation of MOR expression in the trigeminal ganglion. TT replacement therapy could restore the MOR levels in the trigeminal ganglia of morphine-tolerant rats, suggesting an essential role of TT in regulating the function of the peripheral MOR system [75].

## The role of progestin in the sex-biased response to opioid analgesics

Progestins include progesterone, 20α-hydroxyprogesterone and 17α-hydroxyprogesterone, among which progesterone has the strongest biological activity [76]. Progesterone (P4) is commonly produced by the adrenal cortex as well as the gonads, which consist of the ovaries and the testes. P4 is secreted by the corpus luteum of the ovary in early pregnancy and by the placenta in late pregnancy [77]. In the luteal phase of the menstrual cycle, the secretion of P4 by the ovarian corpus luteum is stimulated by high levels of oestrogen [78]. Usually, P4 and E2 have synergistic effects. For example, during pregnancy, the rise curves of these two hormones in the blood are parallel, and the peaks are reached at the end of pregnancy. The strong contraction of the uterus during childbirth is closely related to the synergistic effect of P4 and E2 [79]. It has been demonstrated by both clinical and animal studies that during pregnancy, the threshold for responsiveness to painful stimuli is elevated [80,81]. The elevation in the maternal nociceptive threshold is partly attributed to the decreased function of the endogenous opioid system, which can be mediated by the change in peripheral E2 and P4 during pregnancy [81]. In addition to coacting with oestrogen, P4 alone also has a regulatory effect on opioid analgesia. It was found by Sternberg et al. that a single subcutaneous injection of P4 is able to restore κ opioid-mediated analgesia, which was achieved *via* subcutaneous administration of U50488 in OVX female mice [82]. P4 plays a crucial role in modulating the sexually dimorphic analgesia mechanism that involves κ and δ opioid receptors [83]. P4 increased the expression of δ opioid receptors on spinal cord posterior horn neurons in a rat model of peripheral neuropathic pain [84]. In addition, in a rat model of chronic pain after spinal cord injury, subcutaneous P4 injection caused an increase in KOR expression in the dorsal spinal cord [85] (Table 2). Furthermore, the efficacy of opioids can be regulated by inflammatory cytokines, such as tumour necrosis factor α (TNFα), interleukin-1β (IL-1β) and IL-6. The release of inflammatory mediators from neuroimmune cells is sex hormone dependent. P4 was found to be able to repress the production of pro-inflammatory cytokines from astrocytes and microglia cells [86]. Thus, it is speculated that P4 may enhance opioid analgesia by inhibiting the release of inflammatory cytokines in the central nervous system.

Studies exploring the modulatory effects of progestins alone on opioid analgesia are scarce, partly due to the normally parallel existence and combined action of progestins with estrogens. Nevertheless, the findings that a single administration of P4 regulates opioid analgesia reflect the need for further research assessing the specific modulatory effects.

## Discussion

Sex difference has been recognized as a biological variable. Under particular circumstances, the sex difference in the magnitude of opioid effects upon analgesia can be affected by many factors, including the inherent anatomical differences in the pain-coping neuronal circuit, sex hormones, psychological aspects, social and cultural biases, and genetic diversity [5,87]. The present review highlights the pivotal role of gonadal hormones in opioid-mediated analgesia, with emphasis on elaborating the regulatory effect of oestrogens, androgens, and progestins on the opioid system. As reviewed above, gonadal hormones caused opioid analgesia alteration either by modulating the expression of opioid receptors or by regulating the transduction of opioid signalling at subcellular and synaptic levels. The discrepancy regarding hormone modulation of opioid antinociception was due to the various pain modalities examined, different types of opioid receptors, and different doses of oestrogen used. In general, oestrogen is mostly a negative modulator of opioid analgesia *via* both nongenomic and genomic effects. Androgen receptors function as transcriptional regulators of MOR gene activities, which underlies the androgen-mediated facilitation of opioid analgesia. Many studies have reported the combined effect of oestrogens and progestins, while progestins used alone could promote opioid analgesia by increasing the expression of opioid receptors.

Most of the studies described in this review adopted GDX female or male animals as negative controls and observed the effect of chronic or acute administration of a certain type of hormone on opioid analgesia. This single hormone replacement method helped to eliminate the interference of other hormone effects, but it could not detect the combined action of multiple hormones, nor could it fully mimic the pulsatile and circadian features of endogenous hormone concentrations. It is possible that different hormones interact with each other, and the regulatory phenotype of opioid analgesia can be attributed to the comprehensive action of all types of hormones. In addition, on the basis of our previous research, we proposed that the effect of hormones is more related to their fluctuating paradigm than to the expression level at a specific time point. Future studies are encouraged to take into account the combined effect of and the dynamic changes in each hormone when measuring the effects of gonadal manipulations on the outcome of opioid analgesia.

This review focused primarily on the activational effects of gonadal hormones since the majority of the aforementioned studies adopted adult animals to investigate the regulatory role of each hormone on opioid function. However, as mentioned above, completely different behavioural outcomes may be observed when gonadal manipulations are performed early in the developmental stage and in adulthood. In addition, the activity of gonadal hormones in neonatal organisms is functionally linked to that in adulthood. Therefore, the organizational effect of gonadal hormones, which occurs during the period of sexual differentiation, should be given more attention in future research.

For decades, opioids have been the most preferred drugs for pain management. The use of opioids is often associated with many side effects, including tolerance, dependence, addiction, and abuse. As shown in the present review, gonadal hormones are an obvious target for the investigation of opioid-mediated effects. Factors involved in the underlying mechanisms of gonadal hormones on opioid analgesia are probably potential pharmacological targets for precise opioid analgesia. Further and in-depth explorations of the mechanisms contributing to gonadal hormone regulation of opioid analgesia are underway.

## Conclusion

As shown in the present review, there is considerable evidence that different circulating levels of gonadal hormones play major roles in mediating the sexual dimorphism of opioid antinociception. Gonadal hormones exert crucial effects on opioid analgesia either by genomic action on the expression of opioid receptor genes or by nongenomic mechanisms in regulating the transduction of opioid signalling, the functionality of opioid receptors, and the metabolism of opioids.

One obvious implication of this review is that clinical evaluation of the level and fluctuation of each hormone is needed during opioid treatment. From a public health perspective, understanding the regulatory effects of gonadal hormones on opioid analgesia will have a substantial effect on increasing our knowledge of sex-biased and hormone-related differential prescription of opioids. In other words, we support a sex-specific, hormone level-considering method of opioid use to minimize problematic side effects. More research to elucidate the mechanisms underlying sexual dimorphism in response to opioid treatments is warranted to foster precise and individualized treatment of pain in future clinical practice.

## Data Availability

Data sharing is not applicable to this article as no new data were created or analysed in this study.
